# MAGNESIUM SULFATE AMELIORATES HISTONE-INDUCED COAGULATION DYSFUNCTION AND LUNG DAMAGE IN MICE

**DOI:** 10.1097/SHK.0000000000002263

**Published:** 2023-11-15

**Authors:** Tao Zhong, Jiaqi Zhang, Shanjia Chen, Sainan Chen, Ke Deng, Jianbin Guan, Jingjing Yang, Ronggui Lv, Zhifeng Liu, Yong Liu, Ping Chang, Zhanguo Liu

**Affiliations:** ∗ Department of Critical Care Medicine, Zhujiang Hospital, Southern Medical University, Guangzhou, Guangdong, China; † Department of Intensive Care Unit, Shenzhen Hospital, Southern Medical University, Shenzhen, China; ‡ Department of Medicine Intensive Care Units, General Hospital of Southern Theatre Command of PLA, Guangzhou, Guangdong, China

**Keywords:** Coagulation, histone, anticoagulants, sepsis, endothelial cell, organ damage

## Abstract

**Introduction:** Extracellular histones have been determined as significant mediators of sepsis, which can induce endothelial cell injury and promote coagulation activation, and ultimately contribute to multiorgan failure. Evidence suggests that magnesium sulfate (MgSO_4_) exerts a potential coagulation-modulating activity; however, whether MgSO_4_ ameliorates histone-induced coagulation dysfunction and organ damage remains unclear. **Methods:** To measure circulating histone levels, blood specimens were collected from septic patients and mice, and the relationship between circulating histone levels, coagulation parameters, and Mg^2+^ levels in sepsis was investigated. Furthermore, to explore the possible protective effects of MgSO_4_, we established a histone-induced coagulation model in mice by intravenous histone injection. The survival rate of mice was assessed, and the histopathological damage of the lungs (including endothelial cell injury and coagulation status) was evaluated using various methods, including hematoxylin and eosin staining, immunohistochemistry, immunofluorescence, electron microscopy, and quantitative polymerase chain reaction. **Results:** The circulating histone levels in septic patients and mice were significantly associated with several coagulation parameters. In septic patients, histone levels correlated negatively with platelet counts and positively with prothrombin time and D-dimer levels. Similarly, in cecal ligation and puncture mice, histones correlated negatively with platelet counts and positively with D-dimer levels. Interestingly, we also observed a positive link between histones and Mg^2+^ levels, suggesting that Mg^2+^ with anticoagulant activity is involved in histone-mediated coagulation alterations in sepsis. Further animal experiments confirmed that MgSO_4_ administration significantly improved survival and attenuated histone-mediated endothelial cell injury, coagulation dysfunction, and lung damage in mice. **Conclusion:** These results suggest that therapeutic targeting of histone-mediated endothelial cell injury, coagulation dysfunction, and lung damage, for example, with MgSO_4_, may be protective in septic individuals with elevated circulating histone levels.

## INTRODUCTION

Sepsis is the result of severe infection caused by microbial invasion, thereby leading to a dysregulated systemic host response. Despite advances in defining the pathogenesis of sepsis, it remains a leading cause of death in critically ill patients (1). In sepsis, a dysregulated host immune response causes the acute activation of coagulation, microthrombosis, and platelet (PLT) and coagulation factor depletion, ultimately contributing to disseminated intravascular coagulation (DIC) and multiorgan failure (MOF) with no specific treatment currently available (2). Therefore, we aimed to explore a promising and aggressive therapeutic means of targeting pathological coagulation in sepsis (3).

Recent advances demonstrate that extracellular histones are significant mediators of sepsis, with the ability to induce endothelial cell injury, activate PLTs, and promote thrombin formation, which are responsible for DIC and MOF (4–6). The mouse model of extracellular histone-induced injury is characterized by endothelial damage and excessive coagulation activation, with increased concentrations of tissue factor (TF) and von Willebrand factor (vWf), bearing a strong resemblance to sepsis-associated coagulopathy (2,7). In addition, circulating histone levels are higher in septic patients who develop DIC (6); however, the relationship between circulating histones and laboratory markers reflecting coagulation in septic patients and mice has not been reported. Therefore, antagonizing the coagulation dysfunction and MOF mediated by histone toxicity is probably an effective strategy for the treatment of septic patients.

Magnesium (Mg^2+^) is an essential nutrient that maintains normal cellular physiological activity and homeostasis in the body through more than 300 biochemical reactions (8,9). Previous studies on Mg^2+^ and magnesium sulfate (MgSO_4_) have shown an anticoagulant effect with reduced PLT aggregation, blood clotting, and thrombosis (2,10–12). A retrospective observational study has hinted that low Mg^2+^ levels are associated with active coagulation and inhibition of fibrinolysis in septic patients; however, whether Mg^2+^ is a factor affecting coagulation in sepsis is unclear (13). A randomized controlled trial investigated the impact of MgSO_4_ on coagulation properties in gynecological patients undergoing pelvic surgery and observed that maintaining Mg^2+^ levels at the upper end of the normal range by MgSO_4_ administration attenuated postoperative hypercoagulability (12).

As previously mentioned, MgSO_4_ can affect blood coagulability. However, there are no reports concerning whether MgSO_4_ affects the coagulation system during histone-mediated lethal coagulation abnormalities. Uncovering the role of MgSO_4_ may lead to the development of new therapeutic strategies. Therefore, in this study, we aimed to explore the “MgSO_4_–extracellular histone–coagulation” relationship, preliminarily evaluating the correlation between circulating histone levels, coagulation parameters, and Mg^2+^ levels in septic patients and mice through clinical data and animal experiments, and further confirming whether MgSO_4_ interferes with histone-mediated coagulation abnormalities and organ damage.

## MATERIALS AND METHODS

### Clinical data

Blood specimens of patients diagnosed with sepsis on the basis of SEPSIS-3 were obtained from Zhujiang Hospital of Southern Medical University (14). Inclusion criteria were patients with life-threatening organ dysfunction arising from an uncontrolled host response to infection, and Sequential Organ Failure Assessment (SOFA) score ≥2. Exclusion criteria were as follows: 1) patients younger than 18 years and older than 80 years, 2) patients with solid organ or bone marrow transplant, and 3) patients taking long-term immunosuppressive drugs or with immunodeficiency. For clinical samples stored in the biobank, all septic patients signed an informed consent form, and the experimental design and research were approved by the Institutional Ethics Committee (2023-KY-153-01).

Circulating histone levels in patients were detected using a Human Histone ELISA assay kit (Jingmei Biotechnology, Jiangsu, China) following the manufacturer's guidelines. Data on coagulation parameters (PLT counts, prothrombin time [PT], activated partial thromboplastin time [aPTT], fibrinogen, and D-dimer) and Mg^2+^ levels in the blood of septic patients examined on admission were retrospectively obtained from the laboratory information system. Notably, serum Mg^2+^ tests were not performed on admission in five patients, who were thus excluded from the analysis of the relationship between histones and Mg^2+^ levels.

### Animals

Specific pathogen-free male C57BL/6 mice (aged 8–10 weeks) were kept in ventilated cages at 22°C to 24°C with free access to food and water under normal 12-h/12-h dark/light cycles. Mice were acquired from Guangdong Medical Laboratory Animal Center (Guangzhou, China). Experimental procedures were performed according to international ethical guidelines and authorized by the Institutional Animal Care and Use Committee of Zhujiang Hospital, Southern Medical University (LAEC-2022-071FS).

### Histone-induced coagulation model in mice

To explore the possible protective effects of MgSO_4_, we established a histone-induced coagulation model in mice as previously described (5,7). For survival assessment, mice were randomly allocated into the following four groups: the MgSO_4_ group intravenously received 50 mg/kg of MgSO_4_ (Macklin, Shanghai, China) in the tail vein, the histone group intravenously received 75 mg/kg of histones (Sigma, Santa Clara, CA), the histone + MgSO_4_ group intravenously received histones (75 mg/kg) and MgSO_4_ (50 mg/kg) simultaneously, and the control group intravenously received equal amounts of sterile saline. Regarding drug dosage, it has been reported that 75 and 50 mg/kg of histones are lethal and sublethal doses, respectively, both of which enable mice to achieve circulating histone H3 levels identical to septic patients and mice (7,15). In addition, the MgSO_4_ dose was derived from previous studies, indicating that 50 mg/kg of MgSO_4_ could maintain serum Mg^2+^ levels at the upper end of the normal range but not at toxic levels (12,16).

To evaluate the histopathological damage of the lungs, mice were randomly assigned into the following three groups: the histone group intravenously received 50 mg/kg of histones, the histone + MgSO_4_ group intravenously received histones (50 mg/kg) and MgSO_4_ (50 mg/kg) simultaneously, and the control group intravenously received equal amounts of sterile saline. Three hours after histone challenge, mice were euthanized, and the lungs were harvested for further investigation.

### Cecal ligation and puncture + histone-induced injury model in mice

Cecal ligation and puncture (CLP) was performed as previously described to induce polymicrobial sepsis in mice (17,18). Briefly, mice were anesthetized, and the abdominal midline was dissected under aseptic conditions to expose the cecum. The distal half of the cecum was ligated, and the cecum was then perforated with an 18G needle to extrude a small amount of fecal material from the perforated site. Next, the cecum was placed back into the abdominal cavity, and the peritoneum was closed. All mice were resuscitated by subcutaneous injection of 1 mL sterile saline. CLP mice were randomly allocated into the following three groups: the CLP + histone group intravenously received 20 mg/kg of histones in the tail vein after CLP, the CLP + histone + MgSO_4_ group intravenously received 20 mg/kg of histones and 50 mg/kg of MgSO_4_ simultaneously after CLP, and the CLP group intravenously received equal amounts of sterile saline after CLP. Of note, 20 mg/kg histone can enable mice to achieve circulating histone H3 levels in the range of those found in septic patients and mice (7,15). Blood was collected 24 h after CLP, and laboratory examinations (including coagulation parameters [PLTs, PT, aPTT, fibrinogen, and D-dimer], plasma histone levels, and serum Mg^2+^ levels) were performed. PLT values were measured using a blood cell analyzer BC6000 (Mindray, Shenzhen, China). PT, aPTT, and fibrinogen values were detected on a Sysmex CS5100 (Sysmex, Kobe, Japan). Serum Mg^2+^ levels were examined on a Cobas c702 analyzer (Roche Diagnostics, Mannheim, Germany). According to the manufacturer's guidelines, circulating histone and D-dimer levels in mice were detected using a Mouse Histone ELISA assay kit and a Mouse D-dimer ELISA assay kit (Jingmei Biotechnology), respectively.

### Histopathological analysis

The lower lobe of the left lung in mice was cut and fixed in 4% paraformaldehyde solution for 24 h. The paraffin-embedded tissue was cut into 4 μm thick and stained with hematoxylin and eosin. The severity of lung damage was assessed on the basis of alveolar wall thickening, alveolar congestion, hemorrhage, and inflammatory cell infiltration with a score of 0 to 3, as we previously reported (19). Moreover, freshly obtained samples of the right upper lobe of the lungs were weighed and subsequently put in an oven at 65°C for 24 h and weighed again while they were dried to determine the wet/dry weight ratio (wet/dry weight) and lung water content ([wet − dry weight]/wet [%]).

Fluorescent staining was used for cell injury determination, whereby TUNEL assays were conducted on frozen sections using a commercial kit (Beyotime, Shanghai, China) following the manufacturer's instructions.

### Immunohistochemical staining

Immunohistochemical staining was performed as previously described (20). Paraffin-embedded tissue slides were dewaxed and rehydrated. After blocking with goat serum, each slide was incubated with anti-vWF (ProteinTech Group, Chicago, IL) or fibrinogen-β antibody (Affinity Biosciences, Cincinnati, OH) overnight at 4°C, followed by incubation with corresponding secondary antibody and streptavidin-coupled horseradish peroxidase (Fdbio Science, Hangzhou, China). After washing three times, each slide was visualized after diaminobenzidine (ZSGB-BIO, Beijing, China) staining and hematoxylin counterstaining. Images were captured and analyzed using a digital slide scanner and viewer software (Pannoramic MIDI/Case Viewer 2.3; 3D Histech, Budapest, Hungary).

### Transmission electron microscopy

The tissue was fixed in transmission electron microscopy (TEM) fixative (4°C), followed by fixation with 1% OsO_4_ in 0.1 M phosphate buffer solution for 2 h (20°C–23°C). After gradient dehydration, the tissue was resin embedded, cut into 70 nm thin, and stained with 2% uranium acetate saturated alcohol solution. Subsequently, sections were washed with 70% ethanol and ultrapure water and stained with 2.6% lead citrate. Image acquisition was performed on a transmission electronmicroscope HT7800 (HITACHI, Tokyo, Japan).

### Experimental design of human umbilical vein endothelial cells

We seeded human umbilical vein endothelial cells (HUVECs) at a confluence of approximately 80% per well and stimulated them with histones (100 μg/mL) + MgSO_4_ (0–10 mM) for 1 h to detect the protective effects of MgSO_4_ on HUVECs. The cell viability of HUVECs was measured using the cell counting kit-8 (CCK-8) kit (GlpBio, Montclair, CA). The apoptosis analysis of HUVECs was performed using the annexin V–FITC/PI Apoptosis Detection Kit (Dojindo, Kumamoto, Japan), and the percentage of apoptosis in HUVECs was determined on a CytoFLEX Flow Cytometer (Beckman Coulter, Brea, CA). The extracellular Mg^2+^ levels of HUVECs were measured using a Cobas c702 analyzer (Roche Diagnostics, Mannheim, Germany). To detect the intracellular Mg^2+^ levels, HUVECs were loaded with 2.5 μM Mag-Fluo-4/AM (the Mg^2+^-specific fluorescent dye; Maokang Biotech, Shanghai, China) for 20 min and then subjected to histones (100 μg/mL) + MgSO_4_ (10 mM) for 60 min. The cells were analyzed by a CytoFLEX Flow Cytometer (Beckman Coulter, Brea, CA).

### Quantitative reverse transcription polymerase chain reaction assay

Total RNA was obtained from lung tissue homogenates using AG RNAex Pro Reagent (AG, Changsha, China) following the manufacturer's instructions. Subsequently, cDNA was acquired using Evo M-MLV RT Premix (AG). After mixing the synthesized cDNA, SYBR Green Pro Taq HS Premix (AG), and primers mentioned in Table S1, http://links.lww.com/SHK/B816, quantitative reverse transcription polymerase chain reaction was performed on QuantStudio 3 (Applied Biosystems, Foster City, CA).

### Bioinformatics analysis

To evaluate which biological processes of histones and Mg^2+^ are involved in the pathogenesis of sepsis, bioinformatic analysis was performed (21). Putative targets associated with MgSO_4_ and histones (main pathogenic types: H3 and H4 (5,22)) were harvested from the DrugBank, STITCH, or SuperPred databases. The targets in sepsis were used from web-based GeneCard and DisGeNET databases. The criteria for the selection of sepsis targets in the DisGeNET database were identified as gene-disease association score >0.1, whereas that in the GeneCard database was gene score >1. The correlative targets between MgSO_4_, histones, and sepsis were obtained using Venn diagrams. Subsequently, to explore the underlying biological processes between MgSO_4_, histones, and sepsis, Gene Ontology biological process analysis of the identified correlative targets was performed.

### Statistical analysis

All calculations were performed using GraphPad Prism 8.0 (GraphPad Software Inc., San Diego, CA). Data were expressed as means ± SDs. Discrepancies between groups were evaluated using one-way analysis of variance followed by Tukey's *post hoc* test. Survival studies were explored using the log-rank test. A *P* value <0.05 was considered statistically significant.

## RESULTS

### Circulating histone levels in relation to coagulation parameters in septic patients and mice

**Fig. 1:**
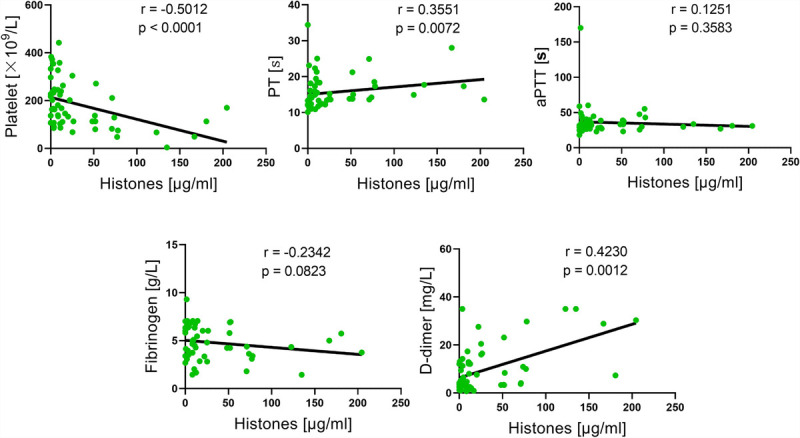
**Association between circulating histone levels and coagulation parameters (PLT counts, PT, aPTT, fibrinogen, and D-dimer) in septic patients (n = 56; Spearman rank correlation)**. aPTT indicates activated partial thromboplastin time; PLT, platelet; PT, prothrombin time.

**Fig. 2:**
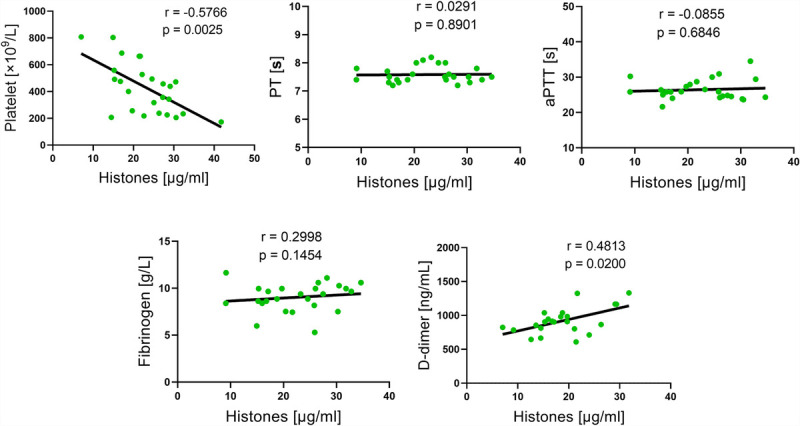
**Association between circulating histone levels and coagulation parameters (PLT counts, PT, aPTT, fibrinogen, and D-dimer) in CLP mice (n = 23–25; Spearman rank correlation).** aPTT indicates activated partial thromboplastin time; CLP, cecal ligation and puncture; PLT, platelet; PT, prothrombin time.

Circulating histone levels are associated with poor clinical outcomes in septic patients (7,23,24). However, the relationship between circulating histone levels and coagulation parameters in septic patients and mice remains unclear. To analyze the relationship between circulating histones and coagulation function, we collected blood specimens from 56 septic patients in the intensive care unit of Zhujiang Hospital of Southern Medical University. The coagulation function was assessed on the basis of several significant laboratory markers, including PLT counts, PT, aPTT, fibrinogen, and D-dimer. The results indicated a significant correlation between histone levels and coagulation parameters in these septic patients, among which histones were negatively correlated with PLTs and positively with PT and D-dimer (Fig. 1). Furthermore, we made similar observations in CLP mice, a widely used septic model, which indicated that histones were negatively correlated with PLTs and positively with D-dimer (Fig. 2). These outcomes propose that histones may be involved in coagulation changes in septic patients.

### MgSO_4_ may play a significant role in histone-induced septic injury through coagulation-related biological process regulation

**Fig. 3:**
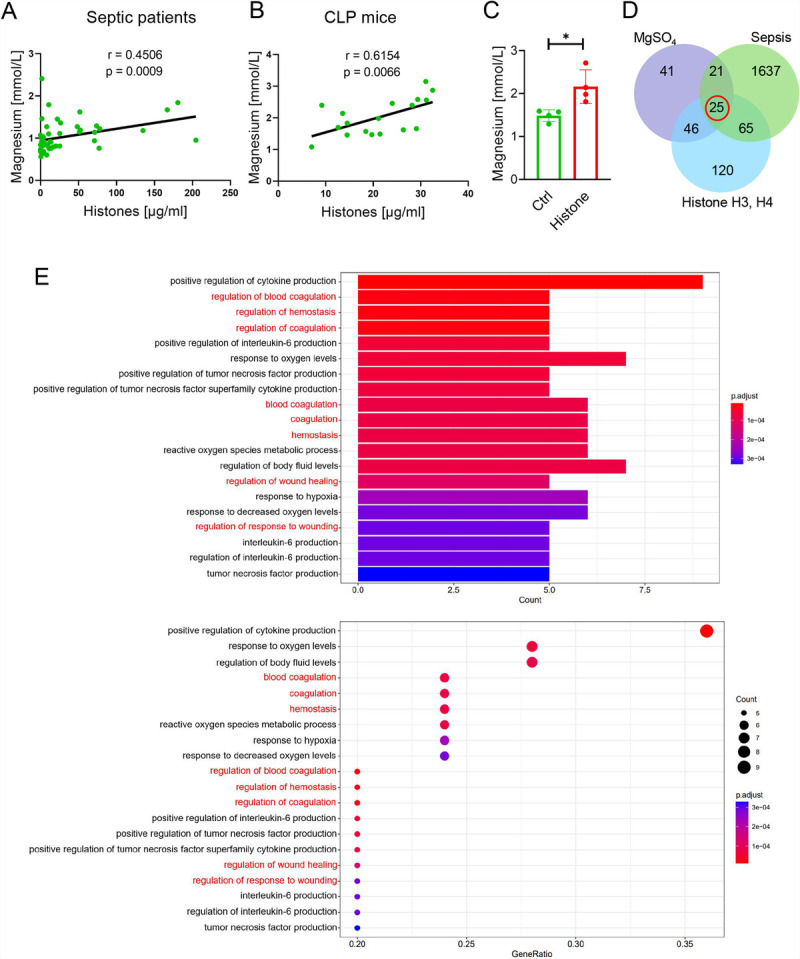
**Mg^2+^ may play a significant role in histone-mediated septic injury through coagulation-related biological process regulation.** A–B, Association between circulating histone and Mg^2+^ levels in septic patients (n = 51) and mice (n = 18; Spearman rank correlation). C, Serum Mg^2+^ levels in mice injected intravenously with 50 mg/kg histones (n = 4). *P < 0.05 versus the control group. D–E, Bioinformatic analysis between MgSO_4_, histones (H3 and H4), and sepsis. D, Venn diagram of the correlative targets between MgSO4, histones, and sepsis. E, Gene Ontology biological processes of correlative targets between MgSO_4_, histones, and sepsis. Red fonts: biological processes related to coagulation.

Our further analysis showed a positive correlation between circulating histone and Mg^2+^ levels in septic patients and mice (Fig. 3, A and B). In addition, serum Mg^2+^ levels were significantly higher in mice injected intravenously with 50 mg/kg histones (Fig. 3C). To evaluate which biological processes of histone and Mg^2+^ are involved in the pathogenesis of sepsis. We determined the correlative targets of MgSO_4_, histones (main pathogenic types: H3 and H4 (5,22)), and sepsis through bioinformatics methods. Further Gene Ontology analysis showed that MgSO_4_ may play an important part in histone-induced septic injury through coagulation-related biological process regulation (Fig. 3, D and E).

### MgSO_4_ protects mice against histone-induced death

**Fig. 4:**
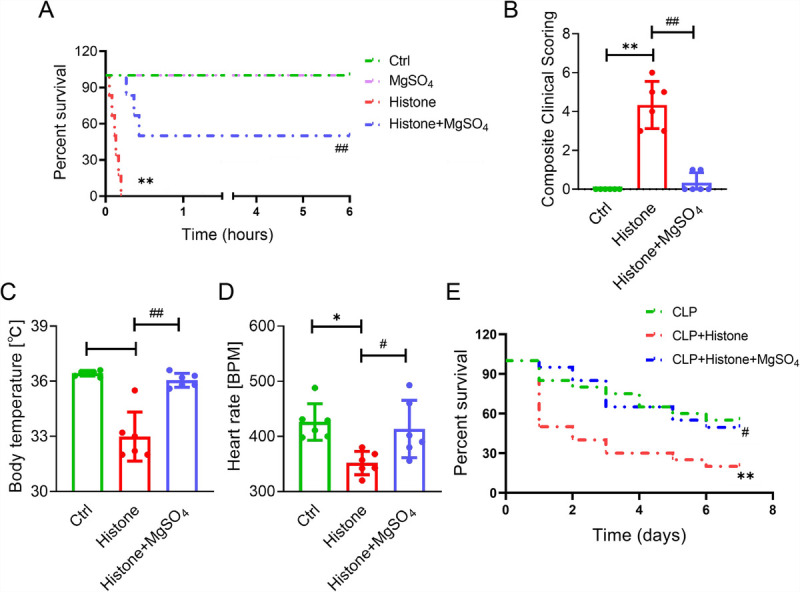
**MgSO_4_ protects mice against histone-induced death.** A–D, Survival rates were determined until 6 h in mice exposed to lethal doses of histones (75 mg/kg) + MgSO_4_ (50 mg/kg). For clinical evaluation, mice were observed for 3 h after stimulation with a sublethal dose of histones (50 mg/kg) + MgSO_4_ (50 mg/kg). A, Survival rate (n = 6). B, Composite clinical score recorded by a double-blinded experimenter according to an index (described in Table S2, http://links.lww.com/SHK/B816; n = 6). C, Body temperature (n = 6). D, Heart rate (n = 6). E, Survival rates were determined until 7 days in CLP mice with or without intravenous injection of 20 mg/kg histones and 50 mg/kg MgSO_4_ (n = 20). *P < 0.05; **P < 0.01 versus the control group. #*P* < 0.05; ##*P* < 0.01 versus the histone group. CLP, cecal ligation and puncture.

We assessed the significance of MgSO_4_ *in vivo* by challenging the mice with an intravenous injection of histones. Consistent with a previous study (7), mice challenged with histones (75 mg/kg, lethal doses) suffered from oronasal hemorrhage and respiratory distress and subsequently succumbed within 1 h, whereas 50% of mice treated with 50 mg/kg MgSO_4_ (histone + MgSO_4_ group) remained alive beyond 6 h (Fig. 4A). After histone stimulation (50 mg/kg, sublethal doses), pathological symptoms of mice were observed using a composite clinical scoring method with a double-blind design (25,26) (Table S2, http://links.lww.com/SHK/B816). Moreover, resistance to histone attack was evident 3 h after MgSO_4_ administration, with a 12-fold decrease in the composite clinical score in mice of the histone + MgSO_4_ group compared with those of the histone group (Fig. 4B). As indicated in Figure 4C and D, MgSO_4_ supplementation significantly inhibited the sharp decrease in body temperature and heart rate, which were maintained at normal levels during histone stimulation. To investigate the effect of MgSO_4_ on histone-induced injury in CLP septic mice, we established a composite model of CLP mice with an intravenous injection of histones. We found that circulating histone levels were significantly higher in CLP mice after intravenous administration of 20 mg/kg histones (Fig. S1A, http://links.lww.com/SHK/B816). In addition, histone stimulation induced decreased survival and abnormal coagulation (decreased PLTs, increased PT and D-dimer) in CLP mice (Fig. 4E; Fig. S1B–F, http://links.lww.com/SHK/B816). As expected, MgSO_4_ supplementation improved histone-mediated death and coagulation abnormalities in CLP mice (Fig. 4E; Fig. S1B–F, http://links.lww.com/SHK/B816). Taken together, these findings support the idea that MgSO_4_ is potentially an effective adjunct for the treatment of histone-induced death.

### MgSO_4_ protects mice against histone-mediated histopathological injury in the lungs

**Fig. 5:**
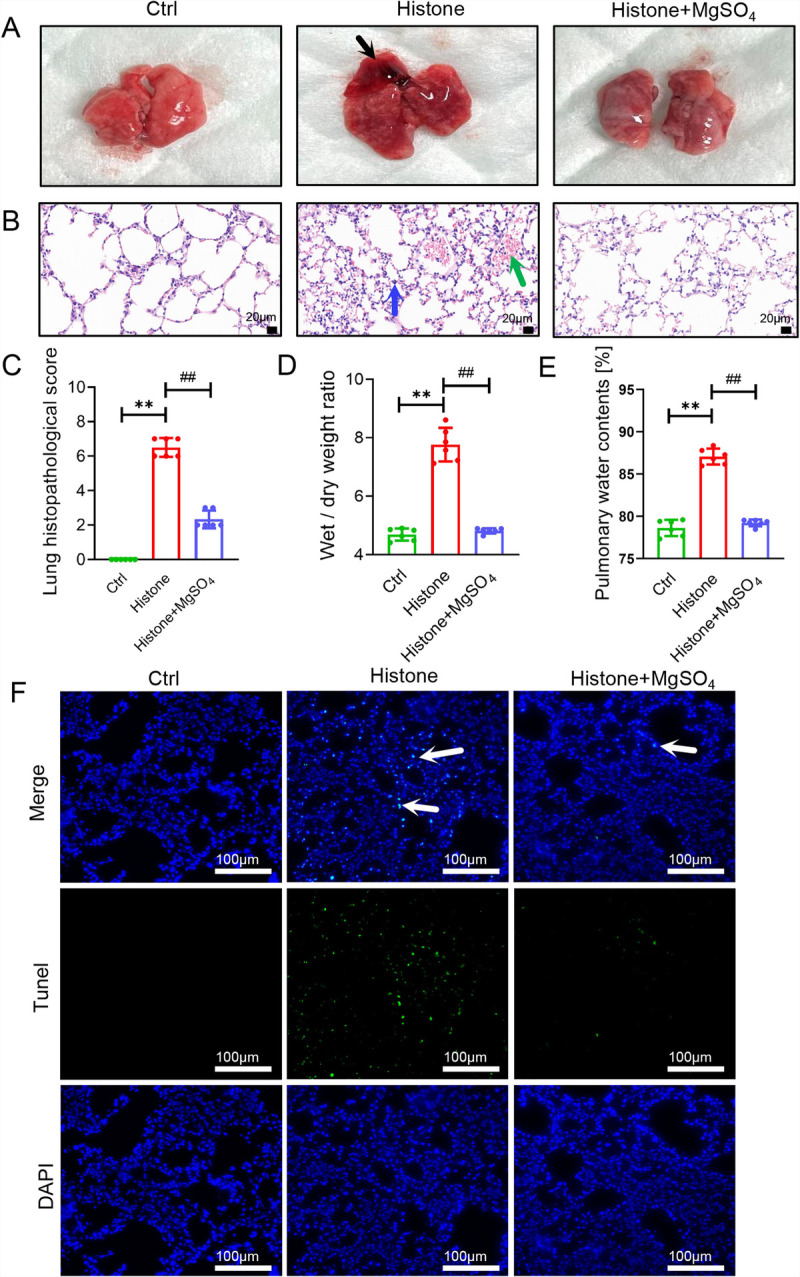
**MgSO_4_ protects mice against histone-mediated histopathological injury in the lungs.** A, Representative gross lung appearance (n = 6). Black arrows: pulmonary congestion and thrombosis. B, Pulmonary HE staining (n = 6). Green arrows: hemorrhage or fibrin deposition; blue arrows: inflammatory cells infiltration. Bar, 20 μm. C, Histological score of the lungs (n = 6). D, Wet/dry ratios (n = 6). E, Pulmonary water contents (n = 6). F, TUNEL staining of the lungs (n = 3). White arrows: positive cell. Bar, 100 μm. **P < 0.01 versus the control group. ##*P* < 0.01 versus the histone group. HE indicates hematoxylin and eosin.

Histopathological analysis revealed that the lungs of MgSO_4_-treated mice had fewer pathological changes, including inflammation, thrombosis, and hemorrhage, than those of mice with only histone stimulation (Fig. 5, A–C). Consistent with these findings, the wet/dry ratio and water content of the pulmonary lobe were significantly lower after MgSO_4_ treatment (Fig. 5, D and E). Furthermore, MgSO_4_ treatment dramatically decreased the abundance of histone-mediated apoptotic cells in the lungs (Fig. 5F). These results indicate that MgSO_4_ can mitigate histone-induced lung damage.

### MgSO_4_ reduces histone-induced endothelium injury and thrombogenesis

**Fig. 6:**
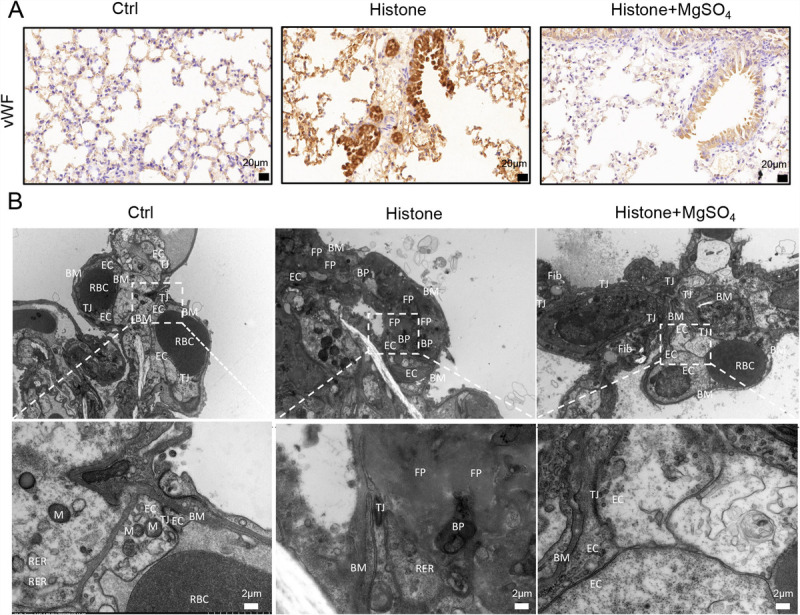
**MgSO_4_ reduces histone-induced endothelium injury and thrombogenesis.** A, Immunohistochemical staining of the von Willebrand factor showed endothelial cell injury (n = 6). B, Morphological manifestations associated with thrombus examined using TEM (n = 2–3). TEM findings of the histone group showed reduced endothelial cells in the lumen of the microvessels, with large amounts of fibrin and PLTs aggregating and obstructing the vessels. The rough endoplasmic reticulum was dilated, and the membrane was broken. BM indicates basement membrane; BP, blood platelet; EC indicates endothelial cell; Fib, fibrocyte; FP, fibrin deposition; M, mitochondrion; PLT, platelet; RER, rough endoplasmic reticulum; TEM, transmission electron microscopy; TJ, tight junction.

**Fig. 7:**
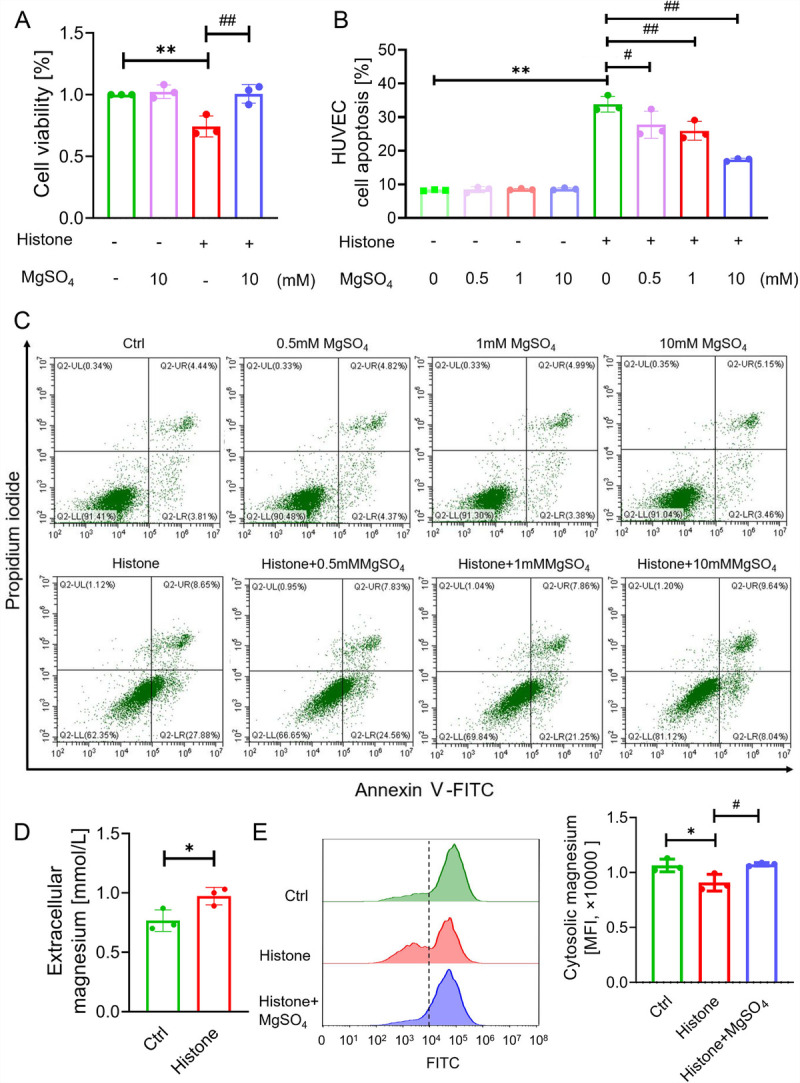
**MgSO_4_ alleviates histone-mediated cell death in HUVECs.** HUVECs were subjected to histones (100 μg/mL) for 1 h with or without MgSO_4_ treatment. A, The cell viability detected by CCK-8 kits. B–C, The proportion of apoptotic cells examined by annexin V–FITC/PI labeling. D, Extracellular Mg^2+^ levels. E, Intracellular Mg^2+^ levels. Results are representative of n = 3 independent experiments. *P < 0.05; **P < 0.01 versus the control group. #*P* < 0.05; ##*P* < 0.01 versus the histone group. CCK-8 indicates cell counting kit-8; HUVECs, human umbilical vein endothelial cells.

To identify the impact of MgSO_4_ on endothelial cell injury and thrombogenesis, the vWF (a significant marker reflecting endothelial cell injury) expression was detected via immunohistochemistry, and thrombus-associated morphological manifestations were examined using TEM. High vWF expression was observed in the lung tissues of the histone group, whereas vWF expression was remarkably decreased upon MgSO_4_ addition (Fig. 6A). Similar to previous studies (7), TEM findings showed that histone stimulation elicited microvascular damage, including reduced endothelial cells, substantial fibrin, and PLT aggregation (Fig. 6B). However, MgSO_4_ treatment significantly reduced histone-induced endothelial cell injury and thrombogenesis. To extend the results *in vivo*, we also explored the protective role of MgSO_4_ against histone-induced cellular damage in an endothelial cell model. To examine the viability of HUVECs, CCK-8 tests and flow cytometry were performed and showed that MgSO_4_ significantly reduced histone-induced HUVECs death in a dose-dependent manner, with as low as 0.5 mM effective (Fig. 7, A–C). Interestingly, we found that histones induced an increase in extracellular Mg^2+^ levels and a decrease in intracellular Mg^2+^ levels of HUVECs (Fig. 7, D-E), which is consistent with previous findings that histones stimulated a significant increase in serum Mg^2+^ levels in mice. However, MgSO_4_ supplementation restored normal intracellular Mg^2+^ levels of HUVECs (Fig. 7E). These data illustrate that MgSO_4_ plays a protective role in histone-mediated endothelial cell injury and thrombogenesis.

### MgSO_4_ ameliorates histone-induced increased TF, fibrinogen, and plasminogen activator inhibitor-1 expression and decreased thrombomodulin expression

**Fig. 8:**
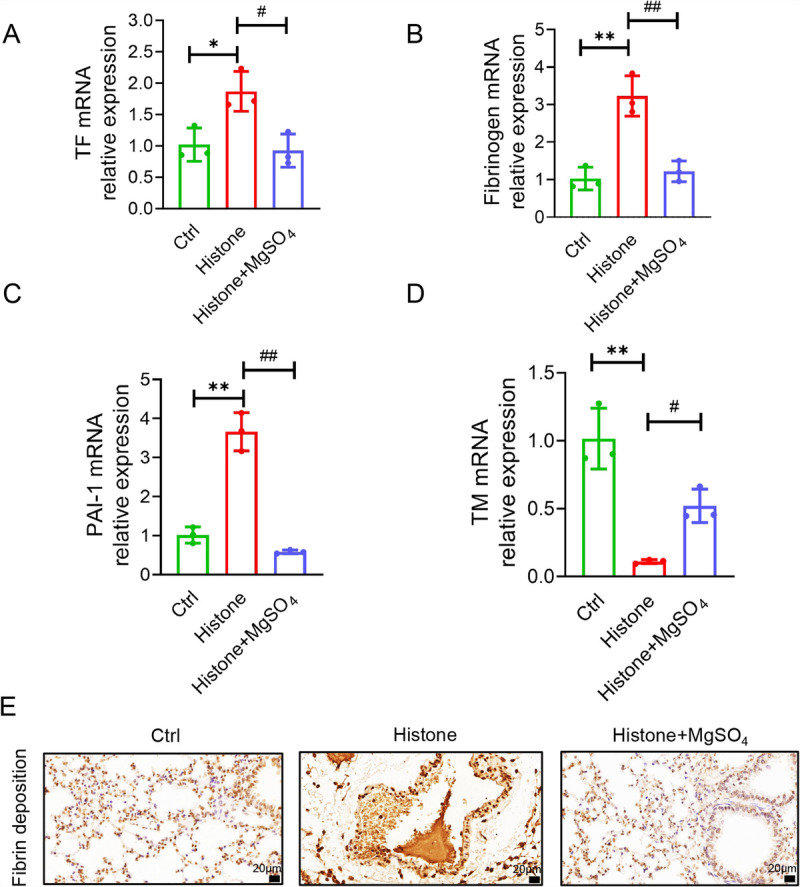
**MgSO_4_ ameliorates histone-induced increased TF, fibrinogen, and PAI-1 expression and decreased TM expression.** A, TF mRNA expression (n = 3). B, Fibrinogen mRNA expression (n = 3). C, PAI-1 mRNA expression (n = 3). D, TM mRNA expression (n = 3). E, Immunohistochemical staining of fibrinogen revealed fibrin deposition and thrombosis (n = 6). Bar, 20 μm. *P < 0.05; **P < 0.01 versus the control group. #*P* < 0.05; ##*P* < 0.01 versus the histone group. PAI-1 indicates plasminogen activator inhibitor-1; TF, tissue factor.

Coagulation and fibrinolytic imbalances, in the form of endothelial damage, coagulation activation, anticoagulation dysfunction, and fibrinolysis inhibition, can lead to extensive thrombosis (27,28). To further illustrate the effect of MgSO_4_ on histone-mediated coagulation and fibrinolytic changes, the mRNA expression of TF and fibrinogen (coagulant), plasminogen activator inhibitor-1 (fibrinolytic inhibitor), and thrombomodulin (anticoagulant) were quantified (Fig. 8, A–D). We observed that TF, plasminogen activator inhibitor-1, and fibrinogen mRNA transcripts were notably increased, and thrombomodulin mRNA transcripts were markedly decreased in the lung tissues after 3 h of histone stimulation compared with those of control mice. However, MgSO_4_ treatment improved histone-induced coagulation and fibrinolytic imbalances. Immunohistochemical findings of fibrinogen (a significant indicator of thrombus status) in the lung tissues further confirmed gene expression results (Fig. 8E). These results showed that fibrin deposition and thrombus formation were extensive in the lung tissues of mice with histone infusion, and MgSO_4_ treatment effectively alleviated fibrin deposition and thrombus formation. Taken together, this study presents a potential therapeutic benefit of MgSO_4_ in histone-mediated coagulation dysfunction and lung damage.

## DISCUSSION

Here, we report that circulating histones in septic patients and mice may be associated with the coagulation process. Intriguingly, we also observed a positive correlation between circulating histones and Mg^2+^ levels in septic patients and mice. Studies have reported an increase in serum Mg^2+^ levels upon exposure to acute stress stimuli (including poisoning, hypotension, and vomiting) (29,30). Therefore, we speculate that serum Mg^2+^ may be related to histone-mediated stress injury in sepsis. Bioinformatic analysis of the “Mg^2+^-histone-sepsis” triad suggests that Mg^2+^ may influence histone-mediated injury in sepsis through coagulation-related biological process regulation. Our further animal studies demonstrate that histones can induce coagulation activation and widespread thrombosis, and MgSO_4_ might offer excellent protection against histone-mediated coagulation dysfunction and lung damage, offering an alternative rationale for treating septic patients with MgSO_4_.

The dysregulated immune response in septic patients can cause coagulation activation, thrombosis, and massive PLT and coagulation factor depletion, which manifest as low PLT counts, prolonged PT, and increased D-dimer concentrations, ultimately leading to DIC and MOF (2,31,32). Extracellular histones have been proposed as significant mediators in sepsis pathogenesis, with several experiments demonstrating that extracellular histones can induce endothelial cell injury and impaired coagulation (5,33). In mouse models, intravenous histone administration provokes perturbations in the coagulation system similar to sepsis-induced consumptive coagulopathy and DIC, including endothelial damage, increased concentrations of TF and vWf, subsequently leading to PLT activation, extensive thrombosis, and coagulation factor depletion, thereby presenting as low PLT counts and prolonged PT and aPTT (2,24,34–36). One study reported that circulating histone levels were higher in septic patients who developed DIC (6); however, the relationship between histones and various significant coagulation parameters (e.g., PLT counts, PT, aPTT, D-dimer, and fibrinogen) remains unclear. In our study, clinical data showed that histone concentrations in septic patients were related to the coagulation process, with histone concentrations negatively correlating with PLT counts and positively with PT and D-dimer values. Furthermore, animal experiments indicated that histone concentrations in CLP mice were also related to the coagulation process, with histone concentrations negatively correlating with PLT counts and positively with D-dimer values. These data suggest that histones may play a significant role in sepsis-induced coagulopathy.

Animal studies have shown that increased serum Mg^2+^ levels occur after acute stress stimulus exposure (20,30). We observed a positive correlation between circulating histones and Mg^2+^ levels in septic patients and mice, and a significant increase in serum Mg^2+^ levels in mice injected intravenously with histones, which might be related to histone-mediated stress damage. Our animal experiments confirm that histones can induce procoagulant reactions and widespread thrombosis, and MgSO_4_ treatment can significantly alleviate histone-induced coagulation dysfunction and lung damage. The possible protective mechanisms of MgSO_4_ are summarized as follows:

1. Excessive endothelial cell injury and subsequent exposed TFs can induce the onset of coagulation dysfunction (24,37,38). Our data showed that serum Mg^2+^ levels were significantly higher in mice injected intravenously with histones. Further experiments confirmed that histones induced a decrease in intracellular Mg^2+^ levels and an increase in extracellular Mg^2+^ levels of endothelial cells, which might then be damaged due to the lack of intracellular Mg^2+^. Studies have shown that exogenous Mg^2+^ supplementation promotes Mg^2+^ influx and increases intracellular Mg^2+^ levels (39–41). Similarly, we observed that MgSO_4_ supplementation increased intracellular Mg^2+^ levels in histone-stimulated endothelial cells. Therefore, increased intracellular Mg^2+^ levels in endothelial cells may maintain cell stability, reduce endothelial cell injury, and ultimately improve coagulation function.
2. Studies have confirmed the role of inflammation in promoting coagulation (13,42). MgSO_4_, as an anti-inflammatory agent (43–45), may modulate coagulation dysfunction by inhibiting inflammation.
3. Calcium (coagulation factor IV) functions as a cofactor throughout the coagulation cascade (13). As a calcium antagonist, Mg^2+^ may regulate coagulation activation by controlling calcium activity (12). As previously mentioned, MgSO_4_ may alleviate histone-mediated coagulation dysfunction and lung damage through several biological activities. However, the underlying mechanisms by which MgSO_4_ ameliorates histone-induced injury have not been well understood and need further investigation.

Previous studies have demonstrated that heparin and recombinant human-activated protein C have antagonistic effects on histone toxicity and can alleviate histone-induced coagulation activation and organ damage (5,7). However, the use of heparin for sepsis may carry a risk of bleeding (24,46). Furthermore, activated protein C is ineffective in the treatment of sepsis and presents a significant risk of bleeding; therefore, it is not recommended for use in septic patients (42,47). Our animal studies have indicated that MgSO_4_ alleviates histone-induced lethal thrombosis and lung damage, and no bleeding adverse effects were observed throughout the treatment. Notably, it has been reported in the literature that Mg^2+^ may regulate coagulation dysfunction and play a protective role in certain bleeding disorders, including intracerebral hemorrhage (48,49). Owing to its versatility, cheapness, and established safety profile as a commonly used clinical drug, MgSO_4_ could be used as a therapy for sepsis (sepsis-induced coagulopathy). A randomized controlled study suggested that MgSO_4_ accelerates lactate clearance in septic patients and may help improve the outcome of sepsis management (50). However, whether MgSO_4_ improves sepsis prognosis and exerts a protective effect by inhibiting histone-induced coagulation dysfunction remains to be further demonstrated.

This study had some limitations. First, it was not designed to investigate the molecular mechanisms by which MgSO_4_ affects histone-induced coagulation dysfunction. Second, we only focused on the short-term effects of MgSO_4_; therefore, long-term effects remain unclear. Third, although MgSO_4_ is effective in improving coagulation dysfunction in histone-induced and CLP + histone-induced injury model mice, its protection against coagulation in other sepsis-related models, such as the endotoxemia model, needs to be further estimated. Finally, although no bleeding was observed during the MgSO_4_ treatment, which was not assessed by laboratory coagulation indicators, MgSO_4_-related adverse effects remain to be further evaluated.

## CONCLUSIONS

These studies are the first to examine the protective effects of MgSO_4_ against histone-mediated injury. Our findings show that MgSO_4_ attenuates histone-mediated coagulation dysfunction and lung damage, suggesting that MgSO_4_ may affect the coagulation system to reduce host susceptibility to histone-mediated death in sepsis. To verify whether proper MgSO_4_ supplementation improves the prognosis of septic patients with elevated circulating histone levels, further studies are warranted.
